# EBCC-15 manifesto: Removing age-related barriers to improve equity in breast cancer care

**DOI:** 10.1016/j.breast.2026.104822

**Published:** 2026-05-27

**Authors:** Fiorita Poulakaki, Etienne Brain, Icro Meattini, Matteo Lambertini, Sophie Pilleron, Tanja Spanic, Francesco Sardanelli, Tania Kalsi, Jennifer E. Kelly, Isabel T. Rubio, David A. Cameron

**Affiliations:** a Europa Donna – The European Breast Cancer Coalition, Milan, Italy; b Breast Surgery Department, Athens Medical Center, Athens, Greece; c Department of Medical Oncology, Institut Curie, Institut Hospitalo-Universitaire des Cancers des Femmes (ANR-23-IAHU-0006) and Inserm U1331, Saint Cloud, France; d Department of Experimental and Clinical Biomedical Sciences “M. Serio”, University of Florence, Florence, Italy; e Radiation Oncology Unit, Breast Unit, Azienda Ospedaliero-Universitaria Careggi, Florence, Italy; f Department of Radiation Oncology (MAASTRO), GROW Research Institute for Oncology and Developmental Biology, Maastricht University Medical Centre+, Maastricht, The Netherlands; g Department of Internal Medicine and Medical Specialties (DIMI), School of Medicine, University of Genova, Genoa, Italy; h Academic Medical Oncology Unit, Ente Ospedaliero Ospedali Galliera, Genoa, Italy; i Ageing, Cancer, and Disparities Research Unit, Department of Precision Health, Luxembourg Institute of Health, Strassen, Luxembourg; j Europa Donna Slovenia, Ljubljana, Slovenia; k Lega Italiana per la Lotta contro i Tumori (LILT) Milano Monza Brianza, Milan, Italy; l Department of Ageing & Health, Guy's and St Thomas' NHS Foundation Trust, London, UK; m CARICE, Faculty of Life Sciences & Medicine, King's College London, London, UK; n Medi-Kelsey Limited, Ashbourne, UK; o Breast Surgical Oncology, Clinica Universidad de Navarra, Madrid, Spain; p Clinica Universidad de Navarra Cancer Center, Madrid, Spain; q Edinburgh University Cancer Centre, Institute of Genetics and Cancer, University of Edinburgh, Edinburgh, UK

**Keywords:** Age, Breast cancer, EBCC manifesto, Elderly, Geriatric assessment, Multidisciplinary team, Personalised approach, Right to be forgotten, Tailored treatment, Young patients

## Abstract

The 2026 European Breast Cancer Conference (EBCC)-15 manifesto provides recommendations aiming to overcome age-related barriers across all stages of the breast cancer pathway (screening, diagnosis, treatment and follow-up). Practical solutions can help to overcome some of the physical and logistical barriers to screening and diagnosis (e.g. out-of-hours and mobile diagnostic services) and clinical trial participation (e.g. patient-centred trial design, reduced complexity and bureaucracy, flexible scheduling, geriatric assessment, age-appropriate physical, psychological and technological support and educational resources). Pragmatic trials should be co-designed with patients and their advocacy groups to address the specific needs of younger and older individuals and fill gaps in our knowledge and understanding (e.g. risk-stratified screening, impact of treatment on reproductive and sexual health, dose optimisation of locoregional and systemic therapy) to avoid under- and over-treatment, adjusting for age-specific needs. Communication training improvements for all healthcare professionals should consider differing age-related priorities. Multidisciplinary follow-up should be tailored to age-related priorities and frailty during and after treatment, including rehabilitation, social and employment support and the right to be forgotten. Decision-making at every stage from screening to post-treatment follow-up should be guided by estimating individual risk rather than by chronological age. Integrating these recommendations into all aspects of care will allow us to offer women with breast cancer the best possible experience and outlook according to each individual's priorities, regardless of their age. More broadly, many of these solutions may be relevant for other cancers with rising incidence in younger and older adults, such as colorectal cancer.

## Highlights

• Specific needs of younger and older women facing breast cancer are often neglected.
• Practical recommendations aim to overcome age-related barriers to optimal care.
• Recommendations encompass all stages of care from screening to long-term follow-up.
• Pragmatic trials should generate evidence to help avoid under- and over-treatment.
• From the outset, include patient advocates in policy, research and implementation.

## Graphical abstract

**Figure:**
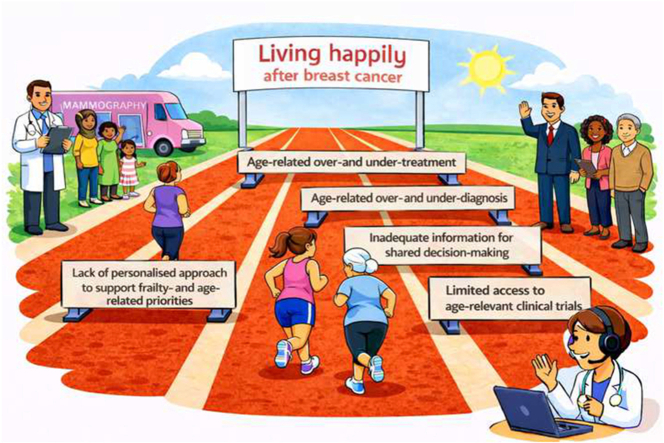

## 1 Introduction

Every 2 years, the European Breast Cancer Council identifies major deficiencies or controversies in the management of patients with breast cancer and selects a multidisciplinary expert team to collaborate in developing principles and recommendations to improve care. At the European Breast Cancer Conference (EBCC)-15, age-related disparities in breast cancer care were considered the highest priority for action. These disparities exist across the entire pathway, from screening and diagnosis to treatment and survivorship care.

In an ageing population, breast cancer incidence and mortality in women aged ≥70 years are expected to almost double by 2040 [1,2]. In parallel, the incidence of breast cancer in young women is also increasing [3,4]. Large-scale data from the USA show increasing rates of metastatic breast cancer across all age groups, but particularly in women aged 20–39 years [5].

**Fig. 1:**
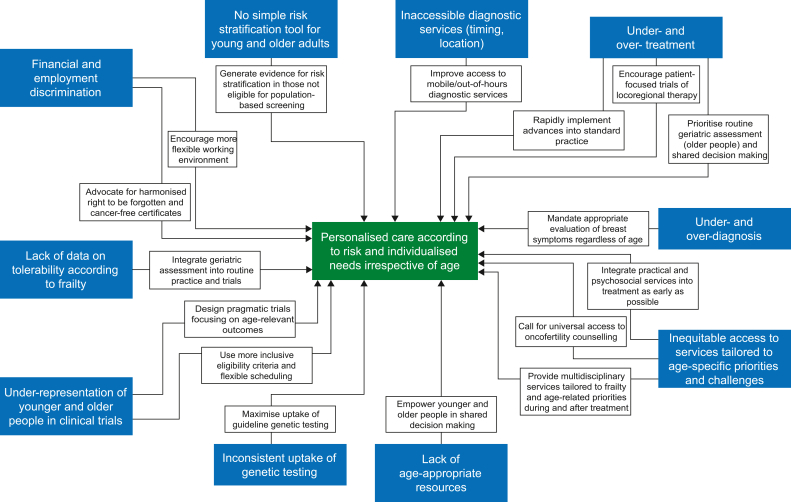
Overview of age-related barriers and recommendations.

This manifesto aims to identify the age-related barriers within each stage of the patient journey and propose solutions that can be implemented across Europe to break down these barriers (Fig. 1). Solutions should be refined and implemented collaboratively with representation from the targeted individuals to ensure relevance and feasibility.

## 2 Methodology

A group of experts, selected by the organising committee of EBCC-15 to represent all stages of the patient journey (patient advocacy [Europa Donna], epidemiology, imaging, radiation oncology, surgery, gerontology, precision medicine and medical oncology), identified and discussed age-related disparities through literature review and frequent virtual meetings and discussion, in the same manner as previous manifesto position papers [[6], [7], [8], [9]]. A medical writer summarised the discussions and collated the recommendations, and all authors critically reviewed all drafts, focusing particularly on their individual areas of expertise, before reaching a consensus on recommendations to overcome age-related challenges. The recommendations were then presented to delegates during a dedicated manifesto session at EBCC-15 and the audience was invited to vote on the recommendations.

While suboptimal treatment in older patients is well documented, there is less evidence in younger women across all stages of the cancer pathway. For the purposes of this manifesto, age ≤40 years was selected to define younger women and age ≥70 years to represent older women, while recognising that individual experiences vary widely and ‘young’ perspectives often extend to women in their 40s facing similar challenges (e.g. fertility, career, parenting). Most of the research described in the literature, particularly for screening, focuses on women. However, as breast cancer in men and women appears more alike than different [10], generally the same recommendations could potentially be extrapolated to men in the absence of evidence specifically in males.

## 3 Clinical trials

Generally, there is less evidence for all aspects of breast cancer management among younger and older than among middle-aged patients because they are often under-represented in clinical trials [[11], [12], [13], [14]], despite some progress towards removing upper age limits. To encourage and facilitate the participation of younger and older adults in clinical trials, efforts should be made to improve workflows, organisation, administration and resourcing of research. In addition, operational and bureaucratic barriers to clinical trial referral should be streamlined or removed (e.g. avoiding lengthy consent forms and trial information requiring many signatures, adopting digital tools enabling personalised provision of information [timing, level of detail] and [preliminary] consent collection) and pragmatic patient-centred trial design should be promoted [[15], [16], [17], [18], [19]]. Accessibility to trials can be improved by re-engineering trial entry criteria to minimise the exclusion of younger or older patients for non-clinical reasons, reducing the complexity of trial procedures, implementing more flexible scheduling and avoiding the need for extra clinic or hospital visits for imaging, blood tests and consultations. For younger patients, flexible schedules accommodating family and work demands should be prioritised. Trial investigators, steering committee members, sponsors (whether academic groups or pharmaceutical companies), regulatory bodies and health authorities share the responsibility to simplify trial participation [17]. They should work together to shape trial procedures that influence acceptance to trial participation and adherence to protocol procedures, and avoid unnecessarily increasing the burden on trial participants. Emotional burden, travel stress, financial toxicity and family disruption must be recognised as real barriers to participation. Overly complex protocols tend to favour enrolment of the ‘perfect patient’ – not only those with excellent performance status, but also individuals advantaged by factors such as race, education, independence, mobility and compliance. It is our collective responsibility to adapt trial processes to the needs of patients, rather than expecting patients to adapt to unnecessarily burdensome trial designs. The collection of patient-reported outcome data can be improved by avoiding redundancy in questionnaires and, where feasible and acceptable, using wearable or digital devices to collect data, potentially assisted by artificial intelligence (AI).

Recommendation 1 Design pragmatic trials considering the specific needs of younger and older people (e.g. competing demands on time, physical and technological access); age-specific trials should ensure collection of characteristics and outcomes most relevant to younger and older women (e.g. reproductive and sexual health, motherhood, geriatric assessment), adjusting for age-related differences in perception and impact of side effects.

• Co-design age-relevant trials with breast cancer advocacy groups from the beginning to align with the needs of both trialists and under-represented populations of patients.
• Facilitate participation of both older and younger adults in clinical trials (e.g. more inclusive eligibility criteria, treatments tailored to frailty, age-appropriate support, more flexible scheduling).

## 4 Screening

Delays in breast cancer diagnosis lead to poor cancer outcomes [20,21]. In most European countries, organised breast cancer screening programmes target women aged 50–69, with 45–74 years as a maximum extension in some countries [22]. However, changes to age limits at which screening is initiated and discontinued have not been made in all European countries, and there is a need to harmonise screening guided by relevant contemporary data.

Some women below or above the national policy screening age may perceive that they are not at risk [2,23]. However, in women aged 40–45 years, cancers detected at the first mammogram are associated with worse survival than those detected at subsequent mammograms [24]. Women ineligible for population-based screening but with other risk factors for breast cancer should not be excluded, and guidelines advocate offering MRI screening to younger women at higher risk [[25], [26], [27]]. Nevertheless, even in specialised clinics, there is substantial variation in breast imaging recommendations for young women at high risk [28]. As formal risk assessment is increasingly adopted in clinical practice, this population should be a priority for future screening imaging studies [28]. It is currently unclear which women outside organised screening programmes should be included, and how evidence to support expanded risk-stratified screening should be generated. Evidence supports breast MRI and mammography in patients with *BRCA* mutations [29,30], and recent data suggest that screening intensity can be tailored according to genetic risk [31]. Risk-stratified screening requires careful implementation addressing ethical, legal and social issues [[32], [33], [34], [35]]. Reducing mortality should be the primary focus, but not the only factor when justifying changes to mass screening programmes or setting priorities for future research. An increase in diagnosis does not necessarily translate to reduced mortality from breast cancer and over-screening should be avoided as it can lead to anxiety and unnecessary medical examinations. Women should receive balanced, understandable information about screening to increase participation.

As breast density decreases with age, screening mammography is more sensitive in older than younger women, resulting in fewer false-positive mammograms and biopsies of benign findings [36]. Furthermore, retrospective studies in older women suggest that detection at an early stage is more likely in screened than non-screened individuals [36,37]. However, these findings are confounded by bias. Moreover, the risk of over-diagnosis (diagnosis of a cancer that would not have caused harm during a person's lifetime) is increased if screening is based purely on chronological age without considering life expectancy, frailty and comorbidities [38,39]. No randomised trials have evaluated screening in women aged ≥75 years [39]. Potential continuation of screening in older women should take into account each individual's health status and personal preference, and should be balanced against competing risks and potential harms [2,40,41]. Prognostic tools are important in guiding the continuation or cessation of screening and assessing the potential of an intervention. However, the limitations of existing tools should be recognised and a more personalised approach should be implemented [42]. Risk should be communicated clearly and sensitively, especially for those with lower health literacy, which may be more prevalent in older people [43].

At both ends of the age spectrum, individualised screening decisions are recommended [2,[44], [45], [46]]. Education and awareness on the importance of breast palpation and self-examination are critical. Personalised screening requires a reliable and accessible tool for risk stratification, accompanied by a simple low-cost approach to implementation. The dynamic evolution of breast density, structure and/or texture over time in previous mammograms may offer an efficient and effective way to estimate breast cancer risk beyond age stratification [47]. The personalisation of screening modalities according to breast density and individualised risk should be considered [[48], [49], [50]]. Furthermore, integrating multigene risk scores into clinical risk models enhances the breast cancer risk calculation [51]. Alternative approaches, such as screening for circulating tumour DNA in breast milk, should be explored further [52]. Prospective investigation of the role of AI-supported screening in younger women is a priority; while the randomised controlled MASAI trial demonstrated increased cancer detection rates with AI-supported screening overall, the effect was not seen in the small subgroup of women aged 40–49 years [53].

Recommendation 2 Maximise uptake of recommended genetic testing and generate evidence to establish and implement personalised breast cancer screening for younger and older women who are not eligible for population-based screening in the context of a precision prevention strategy.

## 5 Diagnosis

Timely primary breast cancer diagnosis is undermined by biases and stereotypes about both younger and older women [[54], [55], [56]]. The assumptions of a lower probability of cancer in women aged ≤40 years [54] and slowly progressing indolent disease in women aged ≥70 years [54,56] can contribute to diagnostic delay and erode patients' confidence in the healthcare system. Despite existing guidelines for young women with breast cancer [14], uptake of recommended genetic testing remains inconsistent across Europe and globally, leaving hereditary risk under-recognised [57,58]. For younger women without classical risk factors, reluctance to report concerns about breast abnormalities may lead to late diagnosis; conversely, healthcare professionals may dismiss concerns in younger women too easily [59], which can lead to diagnosis at a later stage and/or discourage them from reporting future concerns about breast abnormalities.

In symptomatic women, regardless of age, breast cancer diagnosis can be delayed for many reasons, including waiting lists due to limited resources and logistical difficulties in reaching medical centres. Among older women, frailty status, comorbidities, mobility issues, cognitive decline or lack of social support may further limit timely access to diagnostic services [20]. Better access to mobile diagnostic services may be a solution for both younger and older people by providing access to young women who may struggle to balance health needs with work and family demands and to older women who may have functional difficulties, have less social support or rely on working-age family members for support [60]. In addition, collaboration with the primary care sector should be improved, including making GPs aware when a patient has not attended screening.

Recommendation 3 Mandate appropriate evaluation of breast symptoms in all patients regardless of age to avoid under- and over-diagnosis in younger and older adults.

## 6 Treatment

Under-treatment of older adults and under-representation in clinical trials are well documented [2,[61], [62], [63]], with over-reliance on chronological rather than biological age [45]. Locoregional management is critical to breast cancer care and must be personalised according to geriatric assessment and frailty. Radiation therapy is sometimes applied inconsistently, and clinical practice remains fragmented by age-related biases [[64], [65], [66], [67]]. In older fit patients, axillary surgery and radiation therapy are sometimes omitted solely based on age, resulting in a significantly higher risk of recurrence [68,69]. Fit patients with few comorbidities may be denied optimal systemic therapy or be offered suboptimal treatment schedules [70]. Meta-analyses of clinical trials confirm that the benefits of treatment are relative [71,72], and so the *absolute* benefit of a treatment depends on the proportional risk of dying of breast cancer versus another disease. Furthermore, substantial evidence exists on the recurrence patterns of different breast cancer subtypes; therefore, treatment decisions for all patients should incorporate co-morbidities and individualised life expectancy to determine whether the anticipated benefits outweigh the potential risks.

In some older patients, omitting chemotherapy or axillary surgery or tailoring radiation may be appropriate because of comorbidities or frailty, highlighting the importance of geriatric assessment and personalised multidisciplinary management to optimise outcomes [45,[73], [74], [75], [76], [77], [78]]. Older women risk functional decline, reduced autonomy and increased caregiver dependence if treatment decisions ignore frailty or comorbidities. Caregiver burden and the impact of treatment decisions on family members should also be acknowledged and addressed proactively. There is a need to shift clinical decisions from age-based to biology- and frailty-based frameworks through routine geriatric assessment and systematic risk stratification [79]. Furthermore, for older patients, an absence of social support networks should be recorded in medical records, as this may correlate with difficulties in obtaining help with treatment side effects or attending clinic appointments [80].

Breast cancer in younger women can be more aggressive and differs clinically and biologically from disease in middle-aged and older women [14,[81], [82], [83]]. Nevertheless, over-treatment can occur in younger people. There is a tendency to offer younger women the most aggressive and often most toxic combination therapies at the first opportunity, but in some cases this may not be appropriate. For example, young women may receive extended or intensive locoregional therapy (e.g. aggressive upfront multimodality therapy, regional nodal irradiation, boost in all cases) irrespective of disease staging and/or biological risk, with insufficient consideration of long-term adverse effects and despite an unfavourable risk/benefit profile [84]. Aggressive treatment may have a disproportionate impact on health-related quality of life in younger women [85]. Long-term toxicities (e.g. premature menopause, sexual dysfunction, cognitive impairment, chronic fatigue) must be explicitly discussed before treatment decisions are made. Younger patients face body image concerns and disruption of sexuality, family roles and employment, which may be compounded by over-treatment [86,87]. Another consideration is the risk of under-reporting of symptoms (e.g. lymphoedema [88]) or side effects, especially among younger women. Under-reporting may lead to an increased risk of non-adherence to therapy and subsequent poorer outcomes [89].

Both younger and older adults may feel insufficiently informed about the trade-offs between quality of life and efficacy when discussing treatment choice [90,91]. The possible impact on quality of life should be integrated into treatment choice. Furthermore, communication training is essential so that physicians can guide patients through the trade-offs, risks and benefits of treatment [[92], [93], [94], [95]]. Both younger and older adults report unmet needs in communication, including insufficient discussion of risks, benefits and alternatives in language tailored to their age and health literacy [[96], [97], [98]]. Irrespective of age, patients should not be left to navigate complex treatment trade-offs alone. Structured shared decision-making tools and psychosocial support must be systematically embedded in care.

An important contributing factor to under- and over-treatment is the lack of evidence specifically in younger and older patients. Under-representation of older adults in randomised trials perpetuates uncertainty about optimal strategies in this population. On the other hand, proven advances should be integrated into standard clinical practice without delay. For example, there is robust evidence that hypofractionated and partial breast irradiation schedules in older women reduce the treatment burden without compromising efficacy [[99], [100], [101], [102]], yet they are still not consistently adopted in daily practice.

There is an urgent need for more evidence on the best treatments for older women according to frailty [2,103]. Treatment intensity requires adjustment according to each individual's level of frailty or intrinsic capacity, keeping in mind that despite increasing life expectancy, signs of frailty are observed in 40–50 % of people aged ≥70 years diagnosed with early breast cancer and up to 70 % of older people diagnosed with metastatic breast cancer [77,104,105]. Pragmatic trials specifically in older patients are feasible and are important in establishing guidelines for the management of older patients [63,77]. Generating such evidence would allow more representative data to be integrated into algorithms for treatment decision-making. Algorithms based on clinical trial data in highly selected populations (e.g. young or middle-aged adults without any comorbidities) should not automatically be extrapolated to older people, where increasing frailty and competing risks may affect the balance of benefit/risk.

Evidence from pragmatic trials on the balance between de-escalation and escalation strategies in younger and older people is scarce. Questions remain around the potential omission of treatment components, including boost radiation, radiation in low-risk subgroups and endocrine therapy, and trials in these areas should incorporate quality of life and functional outcomes as coprimary endpoints.

Trial designs must integrate quality of life and survivorship concerns alongside oncological outcomes, evaluating treatments in terms of long-term effects on functionality, independence and psychosocial health as well as survival. Data points relevant to older people (e.g. physical function, independence) should be collected routinely in all clinical trials to improve transparency about patient characteristics, and thus the observed impact of a treatment on these important factors. Furthermore, embedded quality of life data collection should use streamlined digital tools to reduce questionnaire burden, but with the option of using more traditional methods if participants prefer. Involving carers can be particularly informative and relevant in older people or those with cognitive impairment or memory difficulties.

Similar to the situation in older patients, there is a lack of evidence on under- and over-treatment among the young. Data relating to breast cancer treatment (particularly tolerability) in younger individuals are scarce [106]. Quality of life-related considerations, such as cosmesis, lymphoedema, fatigue and sexual health, are rarely prioritised when defining locoregional strategies and should be systematically integrated into study designs [107]. Sexual dysfunction is an important issue for all age groups, but especially for the young [108,109]. It is well documented that treatment for breast cancer affects sexual health [110,111], but evidence on the impact of treatment on ovarian function/reserve and fertility, sexual health and pregnancy is limited [112].

In younger women diagnosed with breast cancer who are candidates for systemic anticancer treatment, proper oncofertility counselling is mandatory [113]. Fertility preservation, pregnancy and breastfeeding may be critically important to decision-making and require careful discussion on a case-by-case basis [[114], [115], [116]]. Multidisciplinary pathways should integrate age-appropriate psychosocial services (including sexual health support and comprehensive oncofertility counselling) into the treatment pathway as soon as possible after diagnosis [113,117,118]. Additional efforts are needed to optimise the collection of reproductive outcomes and measurement of ovarian toxicity with all newly developed anticancer therapies [112]. Younger patients face specific pressures from treatment burden, costs, practical challenges such as fitting treatment around work, family and caring responsibilities, returning to work after treatment and psychological issues, including self-image concerns, social barriers, professional challenges and fertility and motherhood concerns [[119], [120], [121]]. Young people facing early-onset breast cancer experience considerable unmet service needs, including inadequate age-appropriate programmes, information needs relating to exercise, recurrence and nutrition, parenting concerns and practical needs for work/school, leading to increased stress [122]. Employment issues may be of greater concern than in older patients; a shorter employment history may be associated with less job security, reduced access to sick pay and fewer opportunities to work from home during treatment. Younger women may require flexible treatment schedules to help balance therapy with work, family and caregiving responsibilities.

Younger women, especially those with dependent children or other family members, may feel considerable emotional and social pressure to accept the most intensive therapy available and continue all work and family commitments [123,124]. Conversely, older women (who may also have caring responsibilities) may feel guilty for choosing or refusing more intensive therapy in later stages of life. These challenges should be queried openly by the care team to help each patient choose what is best for them, regardless of their age. Importantly, prioritising quality of life does not necessarily mean compromising quantity of life in older people, highlighting the importance of individualised value-based care [125]. The content and delivery of information (e.g. mobile apps, online materials, videos, phone calls, printed information) should be tailored to each individual so that it is understandable and accessible.

Recommendation 4 Encourage pragmatic patient-focused trials of locoregional strategies to support changing standards of care for older and younger women.

Recommendation 5 Empower younger and older people in shared decision-making.

• Use age-appropriate educational resources, decision-making tools and information about innovative procedures, treatments and trial participation.
• Provide improved training for healthcare professionals in age-appropriate communication and empathy.

Recommendation 6 For older women with breast cancer, prioritise assessment of biological age through routine geriatric assessment (frailty/intrinsic capacity) and include geriatricians in integrated pathways, decision-making and support infrastructure.

Recommendation 7 Rapidly and consistently implement advances into standard practice and algorithms (including molecular signatures if clinically validated) to balance over- versus under-treatment (e.g. de-escalation in low-risk patients, escalation in selected high-risk patients), which may present differently in older versus younger women.

Recommendation 8 Ensure all patients can access services designed to address their age-specific needs, while recognising individual differences in priorities.

• For younger women, call for universal access to fertility preservation and oncofertility counselling and integrate genetic, practical (caring responsibilities, employment support) and psychosocial (including sexual health support) services into treatment as early as possible.
• For older women, ensure maintained independence through reduced hospital visits, increased care at home if required and awareness of caring responsibilities and social support.

## 7 Follow-up care continuum

Follow-up care must be redefined as a continuum, integrating oncologists, geriatricians (for older women), psychologists, gynaecologists, nurses, primary care physicians and allied health professionals. Most existing clinical practice guidelines focus on prevention and surveillance, with less information on lifestyle guidance, psychosocial support, management of complications, care coordination and psychosocial interventions [126]. Long-term care plans should be developed in collaboration with patients, and should include both medical and non-medical needs (e.g. mental health, financial guidance, return-to-work support). A recent Cochrane review reported that 56–88 % of people diagnosed with breast cancer return to work within 24 months of their diagnosis, and older age and chemotherapy administration may potentially be associated with a lower likelihood of return [127]. More research is needed to understand factors contributing to return to work, so that timely support can be offered as appropriate. A recent French cohort study (CANTO) that adjusted for age, education, family situation and clinical variables showed that working conditions influence return to work: shift work, strenuous work postures, absence of a 48-h work-free period and low involvement in decision-making decreased the likelihood of returning to work 2 years after diagnosis [128]. Employers should be encouraged to understand and accommodate the individual needs of their employees, allowing a flexible and effective return or continuation of work to benefit both employees and employers. A return to (or initiation of) adequate physical activity in survivors is also important, but evidence on the most effective way to encourage sustainable exercise habits is limited [129].

Continued care programmes must tailor interventions by age and frailty, including, for example, cardiac prevention and fall management for older women and fertility counselling, psychosocial support and workplace reintegration programmes for younger women. Programmes should also provide information on how to cope with or mitigate long-term side effects, fear of recurrence, the long-term use of hormone therapy and post-mastectomy considerations [130]. Unmet needs (e.g. information, quality of life, emotional, life perspective) are greater in younger than older patients ≥10 years after diagnosis [131]. Younger women may have specific concerns relating to maternity and breastfeeding [120]. Quality of life monitoring to collect actionable data should become routine, employing digital tools (e.g. wearable or digital devices) to reduce patient burden, while ensuring that digitalisation does not widen inequalities for patients with low digital literacy or limited access to technology. In older women, less frequent surveillance after breast cancer does not appear to increase anxiety [132].

The needs of people with a recent diagnosis differ from those facing the longer-term impact of cancer treatment and may vary according to stage of life and disease. Older women with a recent diagnosis may have more marked dependence on carers. In addition, treatment-related side effects may have different implications for younger versus older women. Follow-up programmes in these specific settings need improvement and should be sensitive to age-related differences.

Finally, discriminative policies for health/life insurance, financial products (e.g. loans, mortgages) and other insurance policies persist long after a breast cancer diagnosis and may have a particularly adverse effect on younger survivors [133,134]. Following effective breast cancer treatment, the ‘right to be forgotten’ and access to a ‘cancer-free certificate’ are of paramount importance, especially for young women who can potentially look forward to a very long and healthy lifetime [135,136]. This issue is particularly relevant for accessing bank loans, mortgages, insurance contracts and employment. Many but not all countries have adopted legal measures to counter financial discrimination; however, only nine EU countries have enacted legislation on the right to be forgotten [133,134,137,138]. Breast cancer survivors in Europe need a co-ordinated effort to end financial and legal discrimination, including a harmonised ‘right to be forgotten’ so that life after cancer is not limited by the past [139]. In some countries, the ‘right to be forgotten’ is earlier in very young people [140]. The number of years before a cancer diagnosis can be ‘forgotten’ varies between countries: when calculating the time after which cancer should be ‘forgotten’, adjuvant hormonal treatment and other targeted adjuvant therapies that are continued long after curative surgery (e.g. CDK4/6 inhibitors, HER2-targeted therapy, immunotherapy) should be excluded. Starting the clock from the time of diagnosis may be a more reasonable approach.

Recommendation 9 Provide personalised multidisciplinary services that provide integrated rehabilitation, psychosocial care and functional support tailored to frailty and age-related priorities for patients during and after breast cancer treatment.

Recommendation 10 Urgently advocate for European adoption of harmonised legislation guaranteeing a ‘cancer-free certificate’ and the ‘right to be forgotten’.

## 8 Conclusions and next steps

Age-related inequalities in breast cancer care affect both younger and older women across the entire breast cancer continuum. Strategies to address these disparities must consider both biological and social determinants of health, including access to timely care, employment protection and access to psychosocial support (Table 1). The active involvement of women and patient advocates throughout all stages of policy development, research design and clinical implementation is essential to ensure that proposed solutions are relevant, feasible and woman-centred. Clinical trials, clinical practice guidelines and post-treatment care pathways should be designed to actively include under-represented age groups, enabling robust, age-specific, evidence-based recommendations. Personalised approaches to screening, diagnosis, treatment selection and follow-up care should replace uniform strategies, recognising the heterogeneity within and between age groups. Finally, European health policies should promote equitable access to innovative diagnostics and therapeutics, comprehensive supportive care, universal provision of fertility preservation services and tailored survivorship resources across each individual's lifespan. By addressing these issues, we can start to break down the age barriers currently faced by younger and older women affected by breast cancer. More broadly, with growing evidence of an increase in early-onset cancers, many of these solutions may be relevant for other cancers with rising incidence in younger and older adults, such as colorectal cancer [141,142]. Equity across age groups is not only a clinical imperative but a matter of dignity, autonomy and fundamental patient rights.

Recommendation 11 At all stages, overcome physical and logistical barriers to optimal care and outcomes regardless of age.

• Improve access to mobile diagnostic services and teleconsultation.
• Schedule appointments around family and work commitments.
• Advocate for a supportive and flexible employment environment, facilitating recovery.
• Provide simple alternatives for those unable or unwilling to use technology.

Recommendation 12 Empower age-appropriate advocacy at all stages of the patient journey (e.g. personalised screening, screening awareness campaigns, educational programmes, psychological support, social and legal assistance).

**Table 1:** EBCC-15 manifesto recommendations.

Clinical trials	1	Design pragmatic trials considering the specific needs of younger and older people (e.g. competing demands on time, physical and technological access); age-specific trials should ensure collection of characteristics and outcomes most relevant to younger and older women (e.g. reproductive and sexual health, motherhood, geriatric assessment), adjusting for age-related differences in perception and impact of side effects	• Co-design age-relevant trials with breast cancer advocacy groups from the beginning to align with the needs of both trialists and under-represented populations of patients • Facilitate participation of both older and younger adults in clinical trials (e.g. more inclusive eligibility criteria, treatments tailored to frailty, age-appropriate support, more flexible scheduling)
Screening	2	Maximise uptake of recommended genetic testing and generate evidence to establish and implement personalised breast cancer screening for younger and older women who are not eligible for population-based screening in the context of a precision prevention strategy	
Diagnosis	3	Mandate appropriate evaluation of breast symptoms in all patients regardless of age to avoid under- and over-diagnosis in younger and older adults	
Treatment	4	Encourage pragmatic patient-focused trials of locoregional strategies to support changing standards of care for older and younger women	
	5	Empower younger and older people in shared decision-making	• Use age-appropriate educational resources, decision-making tools and information about innovative procedures, treatments and trial participation • Provide improved training for healthcare professionals in age-appropriate communication and empathy
	6	For older women with breast cancer, prioritise assessment of biological age through routine geriatric assessment (frailty/intrinsic capacity) and include geriatricians in integrated pathways, decision-making and support infrastructure	
	7	Rapidly and consistently implement advances into standard practice and algorithms (including molecular signatures if clinically validated) to balance over- versus under-treatment (e.g. de-escalation in low-risk patients, escalation in selected high-risk patients), which may present differently in older versus younger women	
	8	Ensure all patients can access services designed to address their age-specific needs, while recognising individual differences in priorities	• For younger women, call for universal access to fertility preservation and oncofertility counselling and integrate genetic, practical (caring responsibilities, employment support) and psychosocial (including sexual health support) services into treatment as early as possible • For older women, ensure maintained independence through reduced hospital visits, increased care at home if required and awareness of caring responsibilities and social support
Follow-up care continuum	9	Provide personalised multidisciplinary services that provide integrated rehabilitation, psychosocial care and functional support tailored to frailty and age-related priorities for patients during and after breast cancer treatment	
	10	Advocate for European adoption of harmonised legislation guaranteeing a ‘cancer-free certificate’ and the ‘right to be forgotten’	
Entire patient journey	11	At all stages, overcome physical and logistical barriers to optimal care and outcomes regardless of age	• Improve access to mobile diagnostic services and teleconsultation • Schedule appointments around family and work commitments • Advocate for a supportive and flexible employment environment, facilitating recovery • Provide simple alternatives for those unable or unwilling to use technology
	12	Empower age-appropriate advocacy at all stages of the patient journey (e.g. personalised screening, screening awareness campaigns, educational programmes, psychological support, social and legal assistance)	

## CRediT authorship contribution statement

**Fiorita Poulakaki:** Conceptualization, Project administration, Supervision, Visualization, Writing – original draft, Writing – review & editing. **Etienne Brain:** Writing – review & editing. **Icro Meattini:** Writing – review & editing. **Matteo Lambertini:** Writing – review & editing. **Sophie Pilleron:** Writing – review & editing. **Tanja Spanic:** Writing – review & editing. **Francesco Sardanelli:** Writing – review & editing. **Tania Kalsi:** Writing – review & editing. **Jennifer E. Kelly:** Project administration, Visualization, Writing – original draft, Writing – review & editing. **Isabel T. Rubio:** Conceptualization, Writing – review & editing. **David A. Cameron:** Conceptualization, Funding acquisition, Writing – original draft, Writing – review & editing.

## Declaration of competing interest

FP reports the following non-financial interests: Founding President of non-profit organisation Build a Bridge Foundation in Athens, Greece; member of the British Breast Group; and past Vice President of Europa Donna – The European Coalition. EB reports honoraria from Pfizer, Lilly and Incyte, consulting/advisory roles for Pfizer, Sandoz-Novartis, Daiichi Sankyo/AstraZeneca and Menarini, and travel/accommodation/expenses from Pfizer and Novartis. IM declares honoraria from Eli Lilly, Novartis, Pfizer, Daiichi Sankyo, AstraZeneca, Gilead, MSD, TEMA Sinergie, Exact Sciences and Menarini Stemline. ML reports advisory roles for Roche, Lilly, Novartis, AstraZeneca, Pfizer, Gilead, MSD, Pierre Fabre, Menarini, Nordic Pharma, Bayer, Ipsen and Daiichi Sankyo, speaker honoraria from Roche, Lilly, Novartis, Pfizer, AstraZeneca, Takeda, Ipsen, Sandoz, Libbs, Daiichi Sankyo, Gilead and Menarini, travel grants from Gilead, Roche and Daiichi Sankyo, research funding (to his institution) from Gilead, and other financial interests (JCO Consultant Editor, Chair of the ESO Certificate of Competence in Breast Cancer) and non-financial interests (member of the executive board of the Breast International Group, member of the Board of the International Society for Fertility Preservation and member of the ASCO Annual Meeting Scientific Program Committee on the Breast Cancer – Local/Regional/Adjuvant Track). SP reports a speaker honorarium from the National Comprehensive Cancer Network. TS reports personal fees for advisory board participation from Roche and for invited speaker engagements at regional conferences from Roche, Pfizer and AstraZeneca (outside the submitted work). FS reports personal fees for sponsored talks or ad hoc advisory board participation from Bayer Healthcare, Bracco Group, GE Healthcare, and Siemens Healthineers and research grants from Bayer Healthcare, Bracco Group and GE Healthcare; in addition, he is the scientific principal investigator of the BreastSCan project (Pan-European Breast Image Platform for Advanced AI-based Breast Cancer Screening), funded by the European Commission under the EU4Health Programme. TK reports speaker/advisory honoraria from Astellas, AstraZeneca, Bayer, ESMO, Janssen, Johnson & Johnson, Merck and Pfizer. JEK reports funding for medical writing support from the European Breast Cancer Council, and fees for medical writing services outside the present work from AGO, Artios Pharma Ltd, BioNTech, Duke Cancer Institute, ENGOT, F. Hoffmann-La Roche Ltd, GEICO, Genentech, GINECO, HECOG, Lilly, MaNGo, NSGO, Pharma& UK, Sera, SOLTI and UNICANCER. ITR reports honoraria from MSD and Novartis for educational lectures. DAC reports non-personal relationships (any fees go to employer) with AstraZeneca, Eisai, Seagen, Roche, Pfizer, Lilly, Novartis, Synthon, GSK, Gilead, Daichi and Menarini/Stemline; he is Chair of the Board of the non-profit “Make 2nds Count” charity and the Breast International Group.
